# Supplementary material for: Are carbon emissions trading and green financial instruments synergistic? -Comprehensive quantitative research based on content analysis
Source: PLoS One. 2024 Mar 7;19(3):e0298601. doi: 10.1371/journal.pone.0298601 (PMC10919601; doi:10.1371/journal.pone.0298601)
Supplement: S1 Data — (ZIP) [file pone.0298601.s001.zip › Data/地级市Z_F工作报告.zip!/┴╔─■/╕╖╨┬╩╨/└┤╫╘╣1⁄2╓┌║┼í╛┬φ┐╦╩2╛▌═°í┐_2008.docx]

各位代表：

　　本届政府于２００３年１月就职，任期即将结束。现在，我代表市人民政府向大会报告工作，请予审议，并请各位政协委员和其他列席同志提出意见。

　　一、过去五年政府工作的回顾

　　市十三届人大一次会议以来的五年，是我市经济转型取得重大阶段性成果的五年，是全市经济社会实现跨越发展的五年，是阜新发生历史性变化的五年。五年来，全市各族人民在中共阜新市委的正确领导下，团结奋斗，励精图治，战胜了前进道路上的诸多困难，谱写了转型振兴和构建和谐阜新的新篇章。

　　一是国民经济进入快速发展的新时期。五年间，全市生产总值由８３．９亿元增加到１９３．６亿元，年均增长１５．６％，分别高于全国、全省５个和３个百分点；人均生产总值由４３５０元增加到１００４０元，突破万元大关。规模以上工业增加值由１８．８亿元增加到４７．７亿元，年均增长１７％。地方财政一般预算收入由５．２亿元增加到１０．９亿元，突破１０亿元大关，年均增长１８．９％。全社会固定资产投资累计完成３９０．９亿元，是上五年的３倍。城市居民人均可支配收入由４６７３元增加到８７００元，农村居民人均纯收入由１５９２元增加到４１５０元，年均分别增长１３％和２１％。

　　二是接续替代产业框架基本形成。坚持以项目为核心，以“工业年”活动为载体，大力推进农产品及食品加工供应、新型能源、煤化工等“三大产业基地”建设，以煤炭为主的单一产业结构逐步向多元化产业格局转变。农产品加工业占全市规模以上工业比重由１２．７％上升到２７．３％，煤炭工业比重由３０．７％下降到１９．５％。装备制造业配套、新型建材、精细化工、新型电子、玛瑙加工、北派服饰等优势特色产业快速发展。五年间，实施投资千万元以上项目４９０个，开工建设了巴新铁路等一批事关长远发展的重大项目，百万千瓦风电城建设全面启动。经过不懈努力，我市被列为国家循环经济试点市。

　　三是农业和农村经济健康发展。坚持城乡统筹，以农民增收为核心，全面启动社会主义新农村建设，农业结构战略性调整取得重大进展。改变了以粮食种植业为主的单一结构，形成了畜牧业、林果业、特色种植业、设施农业四大支柱产业。全面落实了国家关于取消农业税和除烟叶税以外的农业特产税政策，累计发放惠农资金３．９亿元。完成植树造林和封山育林４３５．５万亩，是上五年的３倍，森林覆盖率达到３２．１％，提高１０．４个百分点，生态环境实现了整体改善、局部优化。我市被列入全国防沙治沙示范区。海棠山晋升为国家级自然保护区。

　　四是改革开放实现历史性突破。完成各类国有企业改制３０４户，转制面达８４％。香港力劲、香港百江、大连永盛等２０多个域内外大企业参与国企转制，全市正常生产经营的１２０户地方国有工业企业全部实现转制，焕发了新的生机和活力。平稳实施了王营矿、海州露天矿等煤矿关闭破产工作。民营经济占全市经济的比重逐年提高，成为推进转型振兴的重要力量。坚持扩大规模和提高质量并重，大力推进招商引资，内蒙伊利、香港富年等一批知名企业和重大项目落户阜新。累计招商引资到位２００亿元，年均增长２２．７％；直接利用外资１．１亿美元，出口创汇２．２亿美元，分别是上五年的２倍和４倍。

　　五是城乡面貌发生显著变化。沈彰高速公路通车，阜朝、阜铁高速公路全线开工，阜新至北京的直通旅客列车开通。建设了阿金公铁立交桥、引白水源、污水处理厂等一批重点工程。改造了阜新火车站，新建市长途客运站，新增无人售票公交车１０４台；新建和改造城区街路２１７条，对５５０栋旧楼铺设甬道，７３％的行政村实现通油路。城市绿化覆盖率由３５．１％提高到４０．６％，人均公共绿地由４．６平方米增加到７．９平方米，超过全省平均水平。海州矿排土场土地复垦项目竣工。城市环境空气质量达到国家二级标准天数由１８３天增加到３０４天。“生态园林城市”和“清洁卫生城市”建设取得成效。我市荣获“中国人居环境范例奖”，被评为“水环境治理优秀城市”和省级“园林城市”。

　　六是各项社会事业全面发展。科技进步对经济增长贡献率提高５个百分点。各类教育健康发展。普通高中招生由７０００人增加到１．２万人，高考录取率高于全省１５个百分点；职业教育布局和结构调整成效显著。辽工大办学质量不断提高，新增５个省级重点学科和２个博士后科研活动站。现代京剧《血胆玛瑙》获全国“五个一工程”奖。成功举办了首届中国·阜新玛瑙节。篮球城建设深入推进。我市培养的运动员实现了中国男子赛艇项目世界大赛金牌零的突破。城乡公共卫生和基本医疗服务体系进一步完善，人民健康水平有较大提高。社区服务功能得到加强，９０％以上的办公用房达到省、市规定标准。“友爱阜新”、“平安阜新”建设扎实推进。

　　七是人民生活质量稳步提高。突出改善矿区环境和矿工生活条件，采煤沉陷区治理一期工程顺利完成，１．８万户居民得到安置；实施了全省面积最大的棚户区改造工程，８．２万户居民喜迁新居。１／３以上市区居民告别了沉陷区和棚户区，城市南部和新邱地区人居环境发生翻天覆地的变化。城市人均住房使用面积由１３平方米增加到１７．５平方米。坚持就业优先战略，累计实名制就业２２．３万人，以“零就业家庭”和“４０５０”人员为重点的就业困难群体得到援助，就业矛盾有效缓解。社会保障体系不断完善，城市各类社会保险覆盖面进一步扩大，城乡低保制度逐步健全。全部免除农村义务教育阶段学生学杂费。

　　八是县区经济发展步伐加快。两县五区主要指标增幅均高于全市水平，综合实力明显增强，对全市经济发展起到了重要拉动作用。总量及年均增幅居前三位的是：生产总值，阜蒙县４１．３亿元、彰武县２６．１亿元、海州区１５亿元，新邱区３１．３％、海州区２７．４％、细河区２６．２％；地方财政一般预算收入，阜蒙县１．５亿元、海州区０．９亿元、彰武县０．８５亿元，细河区２７．９％、海州区２３．９％、新邱区２２．１％；全社会固定资产投资五年累计，阜蒙县５３．９亿元、彰武县２９亿元、海州区１７．７亿元，海州区５６．１％、太平区４２％、清河门区３１．８％。

　　九是阜矿集团在转型中做大做强。坚持稳定煤炭生产，加快调整和优化结构，企业管理水平和综合竞争力显著提升，为推进全市转型振兴作出突出贡献。实施了清河门矿、海州立井、五龙矿改扩建工程，新增煤炭生产能力３７５万吨。非煤经济比重由２５％提高到４０％。实施走出去战略，积极推进内蒙古白音华露天煤矿开发。五年间，企业总收入由１８．６亿元增加到５０亿元，年均增长２１．９％；累计上缴税金１０．４亿元，是上五年的１．７倍。

　　各位代表，五年来的发展，使阜新迎来了希望的曙光。全市人民精神振奋，自力更生，埋头苦干，实现了困境中的崛起，求发展、盼富裕的愿望更加强烈，工作热情空前高涨，对阜新的未来发展充满信心！特别是党中央、国务院对资源型城市经济转型更加重视，阜新作为全国第一个试点市，越来越引起国内外的极大关注，中央电视台“和谐中国行·走进阜新”、“对话·东北振兴”等栏目对阜新进行了集中宣传。阜新的知名度和影响力日益扩大，正在成为一座充满生机和活力的城市。

　　各位代表，２００７年，是本届政府任期的最后一年。一年来，我们坚持科学发展，立足关键之年，突出项目建设，全面完成了市十三届人大五次会议确定的预期目标。预计全市生产总值１９３．６亿元，增长１７％，其中，一、二、三产业分别增长１７．３％、１７％和１６．９％；地方财政一般预算收入１０．９亿元，增长２２．６％；全社会固定资产投资１１０亿元，增长８．４％；社会消费品零售总额８７．２亿元，增长１６．１％；直接利用外资和出口创汇分别完成２５００万美元、８１００万美元，分别增长３５．６％和１０１％；城市居民人均可支配收入、农村居民人均纯收入分别达到８７００元和４１５０元，分别增长１４．５％和４５．５％；万元地区生产总值能耗下降４．５％。为全市人民办的１０件实事全部完成。

　　一年来，主要做了以下几方面工作。

　　（一）全力推进“工业年”活动，项目建设取得显著成效。围绕建设“三大产业基地”，全力组织项目攻坚战，一批具有牵动作用的重大项目取得突破性进展。总投资５８．６亿元的巴新地方铁路项目开工建设。总投资４７９亿元的阜新煤化工项目完成总体规划，其中，年产１３亿立方米煤制天然气项目已经省发改委批准立项，年产５０万吨煤制油等３个项目完成可研报告。投资１０亿元的华能一期１０万千瓦风电项目进入安装调试阶段。阜电三期技改、金山煤矸石热电等一批项目竣工投产。

　　华丰食品、德美客肉类加工等一批项目实现当年建设、当年投产。全年新开工投资千万元以上生产经营型项目１６６个，同比增加６０个，其中，北京中电地埋式变压器、太克铸造等２８个项目超过亿元。彰武火电项目可研报告正式上报国家发改委。德尔机械与韩国斗山合资生产柴油发动机项目签订协议。做大做强重点骨干企业，新引进建设永生专用车、香可口杏仁深加工等销售收入亿元以上工业项目３０个，累计达到５０个；新增万达铸业、铃兰味精等销售收入亿元以上企业１０户，达到４０户；新增规模以上工业企业１６户，达到２７５户。阜新发电公司加强管理，实现减亏增盈７０００万元；阜新供电公司积极搞好项目服务，促进了地方经济发展。加强各类园区建设，新增开发面积６５０万平方米。工业经济质量和效益显著提高，规模以上工业实现增加值４７．７亿元，增长２０％；实现利税１４亿元，增长４５％。建筑业完成增加值１０．５亿元，增长９．４％。

　　（二）加大农业结构调整力度，新农村建设迈出新步伐。农业生产克服雪灾、春旱、秋吊等灾害影响，实现粮食总产量３５．２亿斤，增长２９．７％，为历史上第三个丰产年。农业产业化呈现良性发展态势，农产品基地建设取得重要进展。生猪规模化饲养水平高于全省１０个百分点，奶源基地建设与整合成效显著，肉羊、肉驴、白鹅饲养量居全省第一位。动物疫病防控体系建设水平有了新的提高。我市被省政府评为“畜牧倍增计划”先进市。举办了首届中国·阜新肉驴节和第四届中国·彰武白鹅节。设施农业取得新突破，新建日光温室１．２万栋，增长３２％；新建“四位一体”生态模式户３６５０户。“百万农民增收致富工程”扎实推进，新增项目户５．５万户。完成水土流失综合治理２０万亩，新增节水灌溉２．２万亩。机械化保护性耕作达到８２万亩，居全省第一位。人工增雨基地建设得到加强。完成植树造林和封山育林７０．５万亩，集体林产权制度改革转制面达到８５％以上。我市被农业部确定为东北三省唯一的循环农业示范市。扎实推进新农村建设，实施“八大工程”，开展了“百企带百村”、城乡学校医院对口帮扶和新型农民培训等活动。全年投入支农资金２．５亿元，增长１９．２％。

　　（三）积极发展现代服务业，财税金融运行健康平稳。辽西最大的蔬菜批发市场投入使用。时代商业广场和新天地商业广场主体工程完工，水产市场竣工，果品市场开工建设，东市商业区和海州庙周边地区改造工程启动。推进“万村千乡”市场工程，新增各类连锁店２２０家。成功争创“中国优秀旅游城市”，城市形象和竞争力进一步提升。海棠山景区进入国家４Ａ级景区行列。海州矿国家矿山公园建设步伐加快。乡村旅游蓬勃兴起。全市旅游总收入增长８０％。房地产业稳步发展，开发住宅面积２５３万平方米。邮政、通讯、保险等行业有了新的发展。转变理财方式，整合财力资源，保障了重点支出需要。强化审计监督，有效地规范了经济秩序和资金管理。国税收入达到１３．２亿元，增长２２．９％；地税收入突破１０亿元，增长２３．６％。烟草公司、石油公司等单位对全市税收增长作出积极贡献。银行业金融机构实现全行业扭亏增盈，各项贷款余额２０４亿元，比年初净增２６亿元。积极推进企业上市融资，制定出台了《关于支持企业上市工作的若干意见》。

　　（四）启动实施融入战略，对外开放跃上新台阶。适应区域经济发展的新形势，主动融入以沈阳为中心的辽宁中部城市群建设、辽宁“五点一线”沿海经济带开发开放和蒙东地区开发。制定实施了《沈阳、阜新经济与社会合作规划纲要》；彰武打造沈阳“卫星城”工作启动；沈阜两市１０户企业签订长期合作协议，沈阳１５家银行与阜新企业实现对接。与锡林郭勒盟建立了友好市盟，举办了“中蒙六地区”加强战略合作年会。抓住国际产业转移和国内南资北移的有利契机，组织开展１３次境内外招商活动，全市招商引资到位５９亿元，增长２７％。利用外资规模和质量实现历史性突破，中国风电公司投资４７００万美元的风力发电、香港东方公司投资２３００万美元的生物质能源等一批重大项目落户阜新；山东六和集团对阜新大江实施重组并引进日本三井物产加盟，世界５００强企业首次进入阜新。意大利援助阜新矿区老年医院项目正式签约实施。举办了中国光彩事业阜新行、中国·阜新玛瑙博览会、经济转型城市高峰论坛和台商阜新行等活动，引进了苏宁集团等一批知名企业。经济开发区、高新技术产业园区分别完成基础设施建设投资１．３亿元和２２００万元，引进项目实现新的突破。

　　（五）推进体制机制创新，民营经济发展步伐加快。完成各类国有企业改制４３户，其中分类处理壳企业２０户。高德矿关闭破产工作平稳操作。辽宁有线电一厂等３户企业政策性破产工作启动实施。厂办大集体改革准备工作有序推进。组织回购资产经营公司债权３．２亿元，为１０户国有企业改制创造了有利条件。中国储备粮管理总公司和辽宁虎跃快客公司分别对市第三粮库和长客公司实施战略重组。出台了国有企业资产管理办法等一系列政策性文件，规范了国企改制及国有资产评估、转让等行为。注重启动民间资本，不断优化民营经济发展环境。制定了鼓励支持非公有制经济发展的政策措施，组建了中小企业信用担保公司及中小企业信用促进会。民营经济进一步发展壮大，营业收入、增加值、实缴税金均增长２０％以上。

　　（六）充分发挥规划的龙头作用，城市建设和管理水平进一步提高。市中心区、阜蒙县城、新邱区“三位一体”发展规划得到省政府批复。高德公铁立交桥达到通车条件，外环路、五龙立交桥、市南排水主干渠、清河门长途客运站等一批重点工程开工。对振兴路等９８条主干道和街巷路进行了改造。铺设通村油路１５６公里，完成３４个建制镇第二轮规划编制工作。引白水源工程土建及管线铺装主体基本完工。启动实施细河治理二期、九营子河城市段治理和雨污分流工程。污水处理厂试运行，中水回用工程进度加快。改造城市供水管网２０公里，新增供热面积１４１万平方米，新发展煤气用户７０００户。启动创建国家卫生城工作。设立了“环卫工人节”。城市植树４４．４万株，新增城市绿化覆盖面积１００万平方米。新邱矿区排土场治理和海州露天矿地质灾害治理一期工程完工。深入开展“城市建设管理提高年”活动，对市容卫生、交通秩序、环境污染等进行了专项整治。加大住宅小区物业管理力度，出台了加强物业管理工作的意见。继续实施老旧住宅小区改造。积极推进节能减排，阜新发电公司１０万千瓦小火电机组关停，取缔城市中心区４吨以下燃煤锅炉７０余台，城市环境空气质量进一步提高。

　　（七）着力解决关系人民群众利益的突出问题，和谐阜新建设取得新成效。积极做好稳定就业工作，实现就业和再就业７．６万人。继续保持零就业家庭动态为零，一半以上棚户区零就业家庭实现双就业。大力倡导全民创业，实名制扶持创业带头人５５７人。组织城乡劳务输出２６万人次。基本养老保险和基本医疗保险覆盖面分别提高４．４和１５．６个百分点，全市正常生产经营的企业参保率达到１００％。市以下基本养老保险金征缴额为４．２７亿元，增长３０．４％。提高了８．７万名企业离退休人员待遇和１６．７万名城市低保人员保障标准。减免了１．８万城市低保户供暖费。启动了城市低保边缘户救助制度。在棚改新区建立６个帮扶站，对困难群众实施“一站式”帮扶救助。开展“蓓蕾生命救助”和“友爱在阜新，博爱一日捐”活动，对２５００多户低保特困家庭实施医疗救助。棚户区改造一期工程住宅小区配套设施建设基本完成，二期工程全面启动，１０４．８万平方米住宅楼主体完工。采煤沉陷区治理二期工程开工。住房公积金累计归集２．３亿元，２６１４户棚户区和中低收入家庭得到贷款援助。完成城乡危险校舍改造维修２６万平方米，建设九年寄宿制学校６所。对城乡１４．２万名学生实施了义务教育“两免一补”。建立了农村低保制度，６．１万名贫困农民得到救助。社区卫生服务覆盖率达到９８％。新型农村合作医疗参合率达到９５％。由矿总院承担的“阜新农村高盐地区高血压干预防治”省级科技攻关课题取得阶段性成果，６０００余名农民受益。解决了农村７．４万人饮水安全问题，为１１５１户灾民援建了新房。农村改厕率达到５５％。对６２个重点贫困村实施整村扶贫开发、３２０户重点贫困户实施移民扶贫。

　　（八）统筹经济社会发展，各项社会事业全面进步。加强科技创新和成果转化，新增省级高新技术企业５户。全市高新技术产业产值增长２０％。推进校企合作，辽工大组织２００多名教授和研究生深入企业，全面开展促进经济转型科技行动。“环宇牌”橡胶输送带填补了我市没有中国名牌产品的历史空白。推进新一轮“普九”工作，促进教育公平公正、均衡发展。中高等职业教育对外联合办学取得突破。以人力资源大厦为主导的人才市场实现人才交流３．４万人次。继续实施经济转型人才定向培养和“一村一名大学生”工程。农村技术员培训工程启动。阜新蒙医药研究所、彰武县人民医院和５２所乡镇卫生院改扩建工程完工。评剧《东藏圣火》荣获第七届省艺术节金奖，阜新玛瑙雕正式列入国家非物质文化遗产保护名录。举办了“农民工文化节”。市工人文化宫改造一期工程竣工。市体育场全民健身活动中心全面开放。举办了首届“阜新篮球运动大会暨辽宁农民篮球赛”和以迎奥运为主题的“中国·阜新玛瑙围棋文化交流会”。完成１０００个２０户以上自然村通有线电视工作。不断提高人口质量，继续稳定低生育水平。关爱老龄事业，设立了百岁老人津贴。创建了阜新妇女儿童救助基金。残疾人培训中心建成使用。

　　（九）推进民主法制建设，政府自身建设进一步加强。自觉接受人大依法监督和政协民主监督，及时办理人大代表议案、建议和政协提案，办复率１００％，满意率９８％。完善社会治安防控体系，积极推进公安系统“三基”建设，严厉打击各种违法犯罪行为，乡镇、社区警民联防和警务进校园等活动深入开展，人民群众安全感进一步增强。健全应急管理体系，有效应对和处置了２２起突发公共事件。不断完善社区功能，出台了《关于加强和改进社区建设的若干意见》，提高了社区工作人员补助标准。第九届村民委员会换届选举顺利完成。“五五”普法工作深入开展。进一步提高办事效率，共有３４６项行政许可和非行政许可审批项目进入行政服务大厅。全市受理市民投诉２．９万件，办结率８５％。通过市长公开电话及时解决２１０个市民反映的热点、难点问题。认真做好信访工作，坚持市长接待上访制度，解决了１３００多件涉及民生的信访问题。通过《行风热线》为群众和企业解决难题４７１件。发挥市长联络员作用，及时听取群众呼声，推进民主决策。开展劳动关系和谐企业创建活动，维护职工合法权益，累计清理拖欠农民工工资２３００万元。加强廉政建设和行政效能建设，规范和维护市场经济秩序，查处商业贿赂案件３２件。加大行政责任追究力度，查处“行政不作为”等案件１１件。全面落实安全生产责任制，强化专项整治和基础管理，遏制了重大事故的发生，安全生产形势总体保持平稳。

　　认真贯彻《民族区域自治法》，重视和支持民族自治县经济社会发展，民族地区资源开发、特色产业发展步伐加快。依法加强了宗教事务的管理。深入开展双拥工作，提高了现役军人和退伍军人待遇。第二次全国农业普查基本完成。消防管理水平不断提高。物价、档案、地方志、地震监测、外事侨务、对台事务、国家安全、国防教育、民防、民兵预备役等工作进一步加强。

　　各位代表，五年来，全市人民紧紧抓住国家资源型城市经济转型试点市重大机遇，围绕实现产业、环境和人的可持续发展，依靠解放思想、深化改革、扩大开放和科技创新，基本完成了转型振兴第一阶段任务。五年来的实践，我们探索并走出一条符合阜新实际的经济转型之路，为今后的发展积累了经验。主要体会是：必须坚持以科学发展观为统领，紧紧围绕转型振兴这条主线，全面推进经济、社会、文化、政治建设；必须坚持解放思想，强化机遇意识，始终坚持加快发展不动摇；必须坚持走新型工业化道路，注重资源节约与环境保护，推动经济发展方式的转变；必须坚持以项目建设为核心，加快结构调整，构筑多元化产业格局；必须坚持把“三农”问题放在经济社会发展的突出位置，发挥城市对农村的辐射带动作用，大力推进社会主义新农村建设；必须坚持改革开放，推进体制机制创新，全面提高对外开放水平；必须坚持以人为本，更加注重改善民生，切实解决好人民群众最关心、最直接、最现实的利益问题；必须坚持完善社会管理，全力维护社会稳定，为转型振兴创造良好环境；必须坚持统筹兼顾，充分调动方方面面的积极性，努力形成加快转型振兴的整体合力；必须坚持转变政府职能，改进工作作风，不断提高执政能力。

过去五年成绩的取得，是在党中央、国务院和省委、省政府高度重视和亲切关怀下，在市委正确领导下，在市人大、市政协监督支持下，全市各族人民辛勤劳动、团结奋斗的结果。在此，我代表市人民政府，向全市各族人民，向各民主党派、工商联、无党派人士、各人民团体和社会各界人士，向驻阜部队、武警官兵和公安干警，向中省直驻阜单位，向所有关心支持阜新发展的海内外朋友们表示衷心的感谢并致以崇高的敬意！

　　在肯定成绩的同时，我们也要清醒地看到，全市经济社会发展中还存在一些突出问题。主要表现在：经济总量不足，重大项目少，经济发展方式没有根本转变；农业经济结构不尽合理，抗旱能力较弱，农民持续增收的基础不牢固；稳定就业和社会保障压力较大，部分群众生活仍然困难，影响社会稳定的因素还比较多；城市功能仍需完善，污染治理和环境保护力度还需进一步加大；安全生产管理仍需进一步加强；在政府机关中，少数干部缺乏创新意识和进取精神，还不同程度地存在作风不实、效能不高、执行力不强等现象，经济发展软环境需要进一步优化。对于这些问题，我们必须高度重视，在今后的工作中切实加以解决。

　　二、今后五年总体要求和奋斗目标

　　从２００８年起，我们已经进入转型振兴的第二阶段。努力开创阜新转型振兴的新局面，是新一届政府肩负的历史重任。

　　党的十七大报告明确指出：“帮助资源枯竭地区实现经济转型”。国务院近期制定下发了《关于促进资源型城市可持续发展的若干意见》（国发?眼２００７?演３８号），明确提出了建立资源开发补偿机制和衰退产业援助机制、加大财政一般性和专项转移支付力度等一系列政策措施。阜新作为全国第一个资源型城市经济转型试点市，党和国家赋予我市转型试点的地位更加突出、任务更加艰巨、使命更加神圣。我们正面临着前所未有的新优势、新机遇。全市上下要进一步增强发展的紧迫感和责任感，牢牢把握新的战略机遇，积极应对新的挑战，坚定不移地朝着转型振兴的宏伟目标阔步前进。

　　根据市委十届五次全会精神，今后五年政府工作的总体要求是：高举中国特色社会主义伟大旗帜，以邓小平理论和“三个代表”重要思想为指导，深入贯彻落实科学发展观，围绕转型振兴这条主线，进一步深化改革，扩大开放，大力发展以农产品及食品加工、新型能源、煤化工为主的现代工业，以农业产业化为主的现代农业，以物流业、旅游业为主的现代服务业，加快构筑以“三大产业基地”为主体的多元化产业发展格局；坚持资源节约和生态环境保护并重，大力发展循环经济，转变经济发展方式；坚持以人为本，努力扩大就业，完善社会保障体系，加快发展社会事业，促进社会和谐，实现国民经济又好又快发展，为夺取转型振兴新阶段的胜利而努力奋斗。

　　今后五年，要基本完成转型振兴任务。主要奋斗目标是：

　　——在确保质量的前提下，实现主要指标增速高于全省平均水平。到２０１２年，地区生产总值突破５００亿元，年均增长１８％，力争达到２０％；人均生产总值达到２．５万元以上；地方财政一般预算收入达到２３亿元，年均增长１７％以上；全社会固定资产投资累计达到１０００亿元，年均增长２２％；直接利用外资５７５０万美元，年均增长１８．５％；出口创汇１．９亿美元，年均增长１８．７％；万元地区生产总值能耗下降２０％，取水量下降２５％。

　　 ——接续替代产业构建完成，多元化经济格局基本确立。全国重要的农产品及食品加工供应基地基本建成，农产品及食品加工业产值占全市工业总产值３０％以上；全国重要的新型能源基地建设取得重大进展，建成百万千瓦风电城；全国重要的煤化工基地建设初具规模，完成一期工程，启动二期工程。“六大优势特色产业”得到发展壮大。

　　——社会建设加速推进，发展协调性进一步增强。科技事业蓬勃发展，整体教育水平日益提高，文化进一步发展繁荣，基本医疗卫生制度初步建立，广电、体育事业得到更大进步。覆盖城乡的社会保障体系更加完善，劳动力供求基本平衡，历史遗留问题得到更好解决。生态环境得到根本改善。城乡人民生活水平达到全国平均水平，城市居民人均可支配收入达到１．８万元，年均增长１５％以上；农村居民人均纯收入达到８０００元，年均增长１２％以上。

　　完成上述目标，阜新将成为一个接续替代产业完全形成、经济结构基本合理、综合实力显著增强的地区，成为一个人民享有更加充分民主权利、社会文明素质进一步提高的地区，成为一个团结友爱进步、社会和谐稳定、人民安居乐业的地区，成为一个环境优美、市容整洁、生态良好、更加适宜人居的地区，成为一个对外更加开放、形象更加美好、充满生机和活力的地区。

　　三、２００８年政府工作主要任务

　　２００８年，是新一届政府的开局之年，也是转型振兴新阶段的起步之年。全市经济社会发展的主要目标是：实现地区生产总值２３０亿元，增长１８％，力争２０％；人均生产总值达到１．２万元；全社会固定资产投资１３２亿元，增长２０％；地方财政一般预算收入１２．５亿元，增长１３．５％，力争１６．５％；社会消费品零售总额１０２亿元，增长１７％；直接利用外资３０００万美元，增长２０％；出口创汇突破１亿美元，增长２３．５％；城市居民人均可支配收入突破１万元，增长１５％；农村居民人均纯收入４８００元，增长１６％；万元地区生产总值能耗下降４．７％；二氧化硫、化学需氧量排放量分别下降１３％和１６％。

　　（一）深化“工业年”活动，加快产业结构调整，推动经济转型迈出新步伐

　　按照新型工业化的要求，进一步拓展和深化“工业年”活动内涵，用新的理念、新的思路、新的举措谋划和推动工业发展，调整优化产业结构，转变经济发展方式，推进转型振兴向纵深发展。

　　加快建设“三大产业基地”。 巴新铁路上半年要全线开工。全力推进大唐国际阜新煤化工项目核准，力争年内开工。华能风电一期并网发电，开工建设华能二期、辽宁能源、中国风电、沈阳科讯等风电项目，全市风电装机总容量达到６０万千瓦以上；彰武火电项目得到国家核准并开工建设，支持阜新发电公司积极推进四期技改项目前期工作。实施振隆南瓜深加工、新金隆实木地板、维远大豆蛋白等一批农产品及食品加工项目。着力培育优势产业和骨干企业。加快推进德尔公司百万台汽车转向泵和柴油发动机、大金集团新能源一体化以及迪亚公司扩产改造等重点项目；积极引进香港力劲集团铸造项目；开工建设纺织工业园区。支持阜矿集团加快产业结构调整，不断增强整体实力；支持春成集团、阜新双汇、大昌铜业等重点企业做大做强；支持环宇牌橡胶输送带等名牌产品发展壮大。全年新开工建设千万元以上生产经营型项目１８０个，其中亿元以上项目３５个；力争２００个新建、续建项目建成投产。新增销售收入超亿元企业１０户，新增规模以上工业企业４５户。规模以上工业增加值达到６０亿元，增长２２％。

　　深入开展“园区建设推进年”活动。健全园区管理体制和服务体系，进一步完善园区发展规划，科学定位各类园区功能和产业布局。研究制定利益分配政策，围绕产业链延伸，加大招商引资力度，促进特色产业向园区集聚，加快园区主导产业群建设。要多渠道筹资，进一步加大基础设施建设力度，积极为项目入区创造条件，全市新增开发面积和投资千万元以上入区项目均增长４０％。突出抓好经济开发区和高新技术产业园区建设，着力提高入区项目质量、规模和土地利用率。

　　增强企业自主创新能力。加快推进以企业为主体、以辽工大和省农科院等高等院校、科研院所为依托的技术创新体系建设，以消化吸收、再创新为重点，提高自主创新能力，新建企业工程技术研发中心６个，开发重点新产品４２项。大力发展高新技术产业，引导企业运用先进适用技术、信息化手段和现代管理方式改造提升传统产业，高新技术产业产值占规模以上工业比重达到１５％以上。实施名牌战略，加快培育知名品牌和拥有自主知识产权的新产品，支持阜新三沟酒业争创中国驰名商标。

　　（二）加快发展现代农业，扎实推进社会主义新农村建设，努力促进农民增收

坚持走农业现代化道路，把发展现代农业和繁荣农村经济作为首要任务，把农民增收摆在更加突出的位置，统筹城乡经济社会发展，加快社会主义新农村建设，保持农业和农村经济快速发展的良好势头。

　　用工业化理念谋划农业，提高农业产业化水平。鼓励、引导、支持各类资本创办不同类型的农产品加工企业，引进培育一批产业关联度大、带动能力强的龙头企业。大力发展畜牧业，扩大生猪、奶牛、肉鸡、白鹅等配套基地建设规模，坚持规模化、规范化、科学化，重点扶持饲养小区和规模饲养场、户的发展。调整种植业内部结构，抓好特色种植业和优质专用粮生产基地建设。以“菜篮子工程”为重点，整合各类资金，突出抓好设施农业，新建蔬菜保护地１．５万亩。加快发展节水农业、高效农业和以“三杏”、优质梨为重点的林果业生产。积极推进“一村一品”和“一乡一业”，形成一批具有地方特色的农产品品牌。培育集体经济组织等各种形式的经营主体，提高农业集约化程度。加快发展抗灾农业和避灾农业，逐步建立适应干旱气候条件的农业产业结构。

　　突出试点村、镇在新农村建设中的示范作用，努力在基础设施建设、村容村貌整治等方面实现新突破。尊重农民意愿，发挥农民建设新农村的主体作用，加快培养有文化、懂技术、会经营的新型农民。以实施整村推进为重点，将部分贫困村和贫困户优先纳入“百万农民增收致富工程”，新增项目户４万户。多渠道转移农民就业，输出农村富余劳动力１８．５万人次。大力发展县域经济，推进县域工业向规模化、集约化方向发展，鼓励发展乡镇企业，使工业成为县（区）、乡（镇）财政增长、农民增收的主要来源。落实强农惠农政策，不断增加对“三农”的投入。

　　加强基础设施和生态环境建设，改善农业生产条件。大力推进水源、节水灌溉等工程建设，开发利用云水资源。完成水土流失综合治理２０万亩，农田保护性耕作１００万亩。继续加强防沙治沙、农田防护林、村屯绿化等重点工程建设，植树造林和封山育林７０万亩。抓好退耕还林管理和封山禁牧工作，巩固生态建设成果。

　　健全社会化服务体系，提高为“三农”服务水平。以良种工程、科技示范工程为载体，重点推广一批实用技术，加快推进农业标准化生产，增强科技对现代农业的支撑作用。加强动植物疫病防控工作，提高农产品质量安全水平。大力发展农民专业合作组织，加强经纪人职业资格认证培训，提高农民组织化程度。

　　（三）以服务生产和人民生活为出发点，加快发展现代服务业，大力培育新的增长点

　　充分发挥服务业在促进经济结构升级、扩大社会就业等方面的重要作用，把现代服务业放在突出的战略位置来抓，制定规划，完善政策，不断提高服务业发展水平。

　　改造提升传统商贸流通业，构筑现代商业新格局。调整优化商业布局，进一步完善核心商业区整体功能。大力培育建材、家具等专业市场，积极引进国内外知名流通企业。时代商业广场、新天地商业广场投入运营，完成东市商业区改造主体工程、海州庙周边地区改造及水果交易市场改扩建工程，启动蔬菜批发市场二期工程建设。鼓励发展经营业态多样的零售、餐饮等商贸服务，适时启动早餐工程。积极发展农村现代流通方式和新型流通业态，推进“万村千乡”市场建设工程，发展各类连锁店２００家。加强物价监管，稳定市场供应，保障群众生活。

　　依托巴新铁路和高速公路，规划发展物流业。引进和培育一批规模化、专业化、网络化的物流企业，重点推进北方国际物流园等一批物流项目，建设农副产品物流中心，努力打造现代物流节点城市。支持房地产业快速发展，充分发挥其对城市经济的拉动作用。实行部门抓产业责任制，培育会展经济，着力抓好信息、中介及社区服务等新兴服务业的发展。

　　扎实建设“中国优秀旅游城市”，加快发展阜新特色旅游业。进一步改善基础设施，完善服务功能，搞好旅游目的地建设。科学开发和保护旅游资源，重点推进藏传佛教、大漠生态、工业遗产和乡村特色旅游。加快海州矿国家矿山公园建设，搞好海州矿矸石山旅游综合开发。瑞应寺晋升国家４Ａ级景区。加强旅游人才队伍建设，推进经营机制创新，激活旅游市场。

　　（四）以招商引资为重点，全面实施融入战略，进一步提升对外开放整体水平

　　 围绕我市资源优势、产业比较优势，认真研发、包装、储备、吸引一批项目。以长三角、珠三角和日本、韩国、香港、台湾等境内外地区为重点，创新方式，拓宽渠道，紧紧盯住国内外大企业、大集团，着力引进一批知名企业和重大项目。充分发挥企业的招商主体作用，鼓励现有企业与域外大企业挂靠联合、战略重组，实现借势发展。积极打造节会招商平台，全力组织好第二届中国·阜新玛瑙节，力争办成一次规模大、层次高、效果好的经贸盛会。进一步完善招商政策，提高招商质量，切实抓好重大签约项目的跟踪落实，推进项目开工建设。

　　全面融入辽宁中部城市群，加强与沈阳全方位的合作。推进沈阳装备制造业阜新配套基地建设，在交通运输、招商信息、人才培养、融资平台建设、旅游开发网络、文化交流等方面实现与沈阳的资源共享，彰武打造沈阳“卫星城”工作取得实质性进展。积极融入辽宁“五点一线”沿海经济带，在加强与锦州等沿海城市合作中实现加快发展。以巴新铁路、白音华煤田等项目为依托，在资源开发、现代物流业、农业产业化等方面，加强与蒙东地区的经济技术交流与合作。充分发挥巴新铁路的牵动作用，吸引境内外大企业到我市投资。

　　把握国际市场需求，优化出口产品结构。转变外贸增长方式，扩大科技含量高和附加值高的产品出口。以争取国际无偿援助项目和扩大境外劳务输出为重点，进一步开展对外经济技术合作。加强国际劳务人员储备和培训工作，推动对外劳务输出梯次发展。加快推进意大利援助阜新矿区老年医院项目建设。

　　（五）继续深化各项改革，支持民营经济发展壮大，逐步完善转型振兴的体制保障

　　大力推进商贸、交通、建筑等领域国有企业改革，加快国有公益性企业改革。全年完成国企改革２０户。商业、粮食企业改革基本完成，物资企业改革取得实质性进展。继续做好分类处理壳企业工作。对列入国家政策性计划的市电缆厂等３户国有企业启动实施破产。做好厂办大集体改革的各项准备工作。全面加强改制企业履约监管，积极处置和化解国有企业不良债务，妥善处理改制遗留问题，防止国有资产流失。继续完善国有资产监管体系，加强国有产权交易管理，建立国有资产转让、处置监管长效机制，将经营性事业单位国有资产纳入依法监管轨道。积极建立国有资本经营预算制度。

　　 认真落实各项扶持政策，为民营经济提供宽松的发展环境。引导和支持民营企业建立健全现代企业制度，提高经营管理水平和市场竞争能力，促进民营企业向规模化、集团化、品牌化发展。扶持信用担保公司发展，促进中小企业融资，鼓励中小企业组建行业协会，增强自我发展能力。鼓励民营企业增资扩股，引导民间资本投资创办各类企业。加强民营企业经营管理者培训，培养造就一支有现代经营理念、有社会责任感的企业家队伍。民营经济营业收入、增加值、实缴税金均增长２０％以上。

　　 积极推进其它领域改革。扩大农村综合改革试点范围，积极化解乡村债务。按照依法、自愿、有偿的原则，探索建立土地承包经营权流转市场。做好集体林产权制度改革收尾及配套改革，进一步完善农村小型水利工程产权制度改革。树立经营城市理念，盘活城市存量资产，多渠道筹集建设资金。加快事业单位分类改革。建立健全有利于各项社会事业发展的体制机制。

　　（六）加强资源节约与环境保护，大力发展循环经济，增强可持续发展能力

　　把节能减排作为促进科学发展的重要抓手，加快构建以政府为主导、企业为主体、全社会共同推进的工作格局。全面贯彻国家关于节能减排指标体系的统计、监测、考核实施方案和办法。强化企业社会责任，健全激励和约束机制，严格执行新建项目节能评估和环境评价制度，抓好重点耗能企业节能减排。大力推进清洁生产，积极推广先进节能的新技术、新工艺、新产品，逐步淘汰落后生产能力。按照“谁投资、谁受益，谁污染、谁治理”的原则，认真落实责任，把节能减排目标完成情况作为检验经济发展成效的重要标准，纳入政府系统绩效考核，实行一票否决。

　　抓住国家循环经济试点市机遇，大力发展循环经济。规划、实施一批重点项目，提高风能、太阳能、煤层气、生物质能等清洁能源和可再生能源的开发利用水平。鼓励企业循环式生产、产业循环式组合、社会循环式消费，加快培育建设循环经济产业链条和产业园区。进一步提高粉煤灰、煤矸石、矿井水等工业废弃物和伴生资源的综合利用率，推进大鹰水泥低温余热发电、广厦建材粉煤灰砌块和泰山石膏板等一批循环经济项目，加快市循环经济示范区和清河门省级循环经济试点园区建设。以循环农业示范市为契机，逐步建立以农作物秸秆、畜禽粪便、农业副产品、林业废弃物为资源的农业循环经济体系，培育一批利用当地资源形成新能源产业的生态模式村。城市领域要重点搞好建筑、生活垃圾等废弃资源循环利用，提高再生资源回收利用水平。

　　加强污染治理，不断改善生态环境。加大环境保护执法力度，新建项目的环保设施与主体工程要同时设计、同时施工、同时投入生产。突出抓好大气、水和噪声污染等专项整治，实现全年８０％以上的天数达到国家二级以上城市环境空气质量标准。强化环境监察监测，不断提高环境安全应急处理能力。做好第一次全国污染源普查工作。提高水资源的开发、利用、管理和保护水平，努力建设节水型城市。加大勘探找矿力度，加强矿产资源的开发、利用和管理。加快推进海州矿、新邱矿、艾友矿地质环境灾害治理等重点生态建设工程。科学规划、节约集约利用土地。

　　（七）认真做好财税工作，提升金融服务水平，加快企业上市进程

　　壮大现有税源，培育后续税源，千方百计增加财政收入。建立纳税大户激励机制，加大对重点税源项目的支持力度。健全重点税源监控体系，突出抓好矿业、餐饮娱乐、房地产等行业的税收征管，依法清理欠税，确保应收尽收。强化公共财政职能，加强财政预算约束，优化财政支出结构，集中财力求发展、促和谐、办大事。整合财力资源，加强行政事业单位国有资产管理，积极推进“乡财县管”，规范政府采购行为，努力提高财政资金使用效益。严肃财经纪律，加强对重点项目和重点支出的审计监督和财政监督。

　　推进金融生态环境建设。继续开展金融机构业绩评价，不断提升金融服务水平，加大对中小企业信贷投入。支持金融机构加快处置不良资产，防范化解金融风险。推进市商业银行增资扩股和跨区域经营。加快农村金融体制创新。加大金融产业招商力度，积极引进国内外金融和风险投资机构到阜新设立分支机构。逐步建立地方企业信用等级评价制度，改善金融业发展环境。

　　积极引导和鼓励企业境内外上市融资。按照“培育一批、改制一批、辅导一批、申报一批”的方针，加强对上市后备企业的指导和服务，进一步优化企业发展的外部环境，力争实现上市公司零的突破。

　　（八）大力加强基础设施建设，推进“三位一体”规划实施，构筑城市发展新优势

　　认真组织实施“三位一体”规划，拓展城市发展空间。结合产业发展方向，着手编制区域内各分区规划，促进主城区、阜蒙县城、新邱区组团发展，努力构建功能完善、资源共享、联动发展的城市新格局。多渠道融资，加快道路交通、村庄整理改造等基础建设，完成北新路中心段建设。高水平启动新一轮《城市总体规划》修编工作，做好九营子河两岸开发、火车站周边地区改造等重点地区控制性详细规划。

　　实施八大工程，改善城乡环境。一是高速公路建设工程。阜铁、阜朝两条高速公路９月底前建成通车，开工建设彰武至通辽高速公路。二是城乡路网改造工程。打通人民大街与北外环路，完成北外环路与高速公路的连接，对１０条市区街巷路进行改造，完成村通油路２００公里，解决４０个行政村通油路。新建彰武县客运站和４个乡镇客运站。三是地下管网改造工程。提高供热、供气普及率，改造供热主线管网１７公里。四是城市公园改造工程。改造完善人民公园、三一八公园和森林公园。五是城市水系建设工程。推进九营子河治理，加快两岸开发建设步伐。继续搞好细河综合治理。六是水资源综合利用工程。污水处理厂要正式运行，中水回用工程竣工使用。引白水源工程基本达到供水条件，启动城市管网配套设施建设。做好引白水源二期和闹德海水源复线工程前期工作。七是老旧小区改造工程。以解决居民出行和照明为重点，推进老旧住宅小区改造，消灭市区无灯路。八是小城镇建设工程。搞好县城和重点小城镇建设，积极稳妥地推进城镇化进程。

　　强化综合管理，提升城市形象。按照建设“生态园林城市”和“清洁卫生城市”的要求，做好城市街路、社区、游园、广场和出入口以及棚改新区的绿化、美化和亮化，完成植树３５万株，新增绿化覆盖面积１００万平方米。发扬“人民城市人民建、人民城市人民管”的优良传统，动员广大市民积极参与城市管理。健全长效机制，继续开展城市环境综合整治，规范城区车辆停放秩序和散装建筑材料运输，解决好群众反映的热点难点问题，维护良好的交通、卫生、市场环境。深入开展国家卫生城创建工作。完善住宅小区物业服务政策措施，强化以棚改新区为重点的物业管理。大力发展城市公交事业，畅通市区交通，方便市民出行。

　　（九）加快以改善民生为重点的社会建设，完善社会管理，促进社会公平正义

　　实施扩大就业的发展战略，促进以创业带动就业。以稳定就业为目标，全年实现实名制就业４．９万人。加大就业援助力度，保持零就业家庭动态为零，确保有就业能力和需求的棚户区回迁下岗失业人员６０天内实现就业，安排“４０５０”人员就业２０００人。进一步提高劳动者素质，完成普惠制培训４万人。完善政策措施，鼓励全民创业，成立创业指导中心，实名制扶持创业带头人２００名。加强公益性岗位的管理，切实发挥其在公共服务、城市管理等方面的积极作用。加快建立覆盖城乡居民的社会保障体系。强化社会保险扩面，加大社会保险基金征缴力度。启动城镇居民基本医疗保险，着力解决困难企业和关闭破产集体企业退休职工参加基本医疗保险问题。进一步完善城乡低保制度，健全医疗救助等社会救助体系。为１０００户农村特困户援建新房。对５０个重点贫困村实施整村扶贫开发。加强社会福利机构建设，建成市儿童福利院。全面完成棚户区改造二期工程和采煤沉陷区治理二期工程。加强住房公积金的归集和管理。启动经济适用房和廉租房建设工程，逐步解决城市低收入家庭住房困难问题。加强供热运行管理，严格执行供热质量不达标退赔细则，切实维护群众利益。以为民、便民、利民为宗旨，以生活、卫生、文体、平安服务为重点，加快建设社区“十分钟服务圈”。提高职工最低工资标准和企业离退休人员待遇。建立企业职工工资增长集体协商机制和支付保障机制，逐步提高收入水平。认真贯彻《劳动合同法》，发展和谐的劳动关系，努力保障劳动者合法权益。

　　优先发展教育。坚持育人为本、德育为先，全面推进素质教育。积极做好新一轮“普九”工作，促进义务教育均衡发展。加快发展职业教育，重点培育一批适应经济转型和新农村建设的职业技术人才。支持辽宁工大提升办学水平，加快发展步伐，建好职业技术学院。鼓励和规范社会力量兴办教育。加强教师队伍建设，重点提高农村教师素质。建立教师流动制度，加强薄弱学校建设。进一步改善办学条件，改造维修校舍１０万平方米，建设九年寄宿制学校２所，改造棚户区学校８所。全部免除义务教育阶段城市学生学杂费和农村学生书本费。发展医疗卫生事业，提高全民健康水平。加强覆盖城乡居民的公共卫生服务体系建设，将符合条件的社区卫生服务机构纳入城镇基本医疗保险定点范围，新增农村甲级村卫生室１００所，新型农村合作医疗参合率和城市社区卫生服务覆盖面均达到１００％。提高医院经营管理和医疗服务水平，加强医德医风建设，为人民群众提供优质、便利的医疗服务。继续推进农村饮水安全工程，解决７．８万人饮水安全。进一步做好人口和计划生育工作，提高出生人口素质。

　　实施人才强市战略，加强以高层次和高技能人才为重点的各类人才队伍建设。做好规划，创新机制，加强各类人才的培养与引进，营造引得进、留得住、用得好的人才环境。继续推进新一轮“一村一名大学生”培养和农民技术员培训工程，做好引智工作，办好各级各类人才市场，优化配置人力资源。

　　把完善社会管理作为改善民生和促进社会和谐的重要任务，推进“平安阜新”建设。统筹协调各方面利益关系，有效预防和化解各类社会矛盾。全面落实信访工作领导责任制，完善信访工作制度，健全政府主导的维护群众权益机制。强化安全生产管理和监督，进一步落实安全生产责任制，切实抓好重点领域和薄弱环节的专项整治，坚决遏制重特大事故发生。进一步完善突发事件应急管理机制，提高保障公共安全和处置突发事件的能力。加强社会治安综合治理，抓好公安系统“三基”建设，健全治安防控体系，加大对各种刑事、经济违法犯罪打击力度，不断增强人民群众安全感。

　　（十）培育文明风尚，促进文化建设和繁荣，积极发展社会主义民主政治

　　大力建设社会主义核心价值体系，广泛开展群众性精神文明创建活动，深入推进“友爱阜新”建设。加强社会公德、职业道德、家庭美德、个人品德建设，形成男女平等、尊老爱幼、互爱互助、见义勇为的良好社会风尚。加强校园周边环境整治，推进未成年人思想道德建设。

　　加快建立覆盖全社会的公共文化服务体系。重点抓好农村文化信息资源共享工程、乡镇文化中心及社区文化室等公益性文化基础设施建设，着力丰富城乡群众文化生活，不断满足人民群众日益增长的精神文化需求。加强对阜新历史文化和民族、宗教以及民间特色文化资源的挖掘和保护。推进文化创新，繁荣文化市场，大力发展动漫创意等文化产业，增强城市文化实力。支持《东藏圣火》参加全国评剧节。完成工人文化宫整体改造。筹建传媒大厦。完成２．５万户、２０户以上自然村有线电视入户工程。广泛开展以迎奥运为主题的全民健身活动，加快篮球城建设，办好首届中国·阜新篮球节。建设集体育、会展、商贸于一体的高水平、多功能的体育文化中心。做好第二次全国经济普查。大力发展老龄事业，促进妇女儿童工作，关心残疾人生活，发展红十字和社会慈善事业，加强外事、侨务、对台、民防及国家安全工作，不断巩固军政军民团结，扎实推进国防后备力量和双拥工作取得新成果。

　　贯彻依法治国基本方略，加强民主法制建设。自觉接受人大的依法监督和政协的民主监督，认真办理人大代表议案、建议和政协提案。加强与各民主党派、工商联、无党派人士的联系，重视并有效采纳其意见和建议。充分发挥工会、共青团、妇联、科协等组织的作用。坚持和完善基层群众自治制度。加强以村务公开、民主管理为主要内容的农村基层政权建设，积极推进农村社区建设试点。认真组织好第七届社区居委会换届工作。继续开展“五五”普法工作，形成自觉学法守法用法的社会氛围。

　　全面贯彻党的民族政策，落实《民族区域自治法》，巩固和发展平等、团结、互助、和谐的社会主义民族关系。以办好阜蒙县５０年县庆活动为契机，全面提升自治县综合实力水平。依法管理宗教事务，充分发挥宗教界人士和信教群众在促进经济社会发展中的积极作用。

　　四、持之以恒地建设对人民高度负责的政府

　　 转型振兴新阶段对新一届政府提出了新的、更高的要求。各级政府要进一步解放思想，开拓创新，全面提高管理水平和服务水平，努力建设对人民负责、为人民服务、受人民监督、让人民满意的政府。

　　一是坚持解放思想，推进工作创新。要认真学习十七大精神，深入开展“认识新优势，抢抓新机遇，实现新发展”学习实践活动，推动思想解放和观念创新。各级干部要加强学习，不断提高自身素质和能力，适应新形势下发展的需要。要深化对社会主义市场经济规律的认识，充分发挥市场在资源配置中的基础性作用。要创新工作思路和方法，把创新贯穿于每一个系统、每一个环节，着力解决影响和制约科学发展的突出问题。要创新机制，加快形成权责一致、分工合理、决策科学、执行顺畅、监督有力的行政管理体制。要强化忧患意识，进一步增强加快发展的使命感和责任感，以昂扬向上、奋发进取的精神状态，推进经济转型与振兴。

　　二是改进工作作风，优化发展环境。要强化社会管理和公共服务，不断完善政务公开制度，增强政府工作的透明度和公信力。加强电子政务建设，提高市长公开电话、市长接访、市长信箱工作质量，办好《行风热线》节目，进一步完善群众诉求机制。严格行政执法程序，规范执法行为。继续减少和规范行政审批项目，全面简化审批程序，提高市、县行政服务大厅的服务水平，为广大投资者提供全方位优质服务。切实提高各级干部的责任意识，深入开展“行政不作为、行政滥作为”专项治理活动，坚决查处损害投资者利益的案件，不断优化以企业为核心的经济发展环境。要增强大局意识，正确处理部门利益与整体利益的关系，形成工作合力。

　　三是强化绩效考评，提高行政效能。要进一步健全狠抓落实的科学管理机制，从工作成效、工作创新、工作纪律等多方面建立健全绩效考核评价机制，将县区纳入绩效考核范围。研究制定办法，加强对考核结果的应用，使考核真正起到鼓励、鞭策、教育作用。加强行政效能监察，全面推行以行政首长为重点的行政问责制，提高各级政府工作质量和效率，确保政令畅通，切实增强执行力。要通过调研、咨询、听证、公示、专家论证等方法，形成科学民主的决策机制，提高决策水平。进一步精简会议，减少文件，规范各种检查、评比和验收活动，降低行政成本。

　　四是加强反腐倡廉，坚持从严治政。以完善惩治和预防腐败体系为重点，进一步落实党风廉政建设责任制，严肃查处滥用职权、贪污贿赂等违法违纪案件。以规范权力运行、资金监控和从政行为为重点，强化对项目招投标、行政权力使用、国有资产转让等环节和部位的制约和监控，加强对领导干部经济责任的审计监督和重特大责任事故的责任追究，确保权力阳光运行。深入开展专项治理，继续纠正教育、医药、医疗等领域的不正之风，着力解决在土地使用、工程管理、社保基金等方面损害群众利益的突出问题。

　　各位代表，我们已经迈进一个崭新的发展阶段。站在新的历史起点，新一届政府肩负着开创转型振兴新局面的历史使命。我们坚信，有中共阜新市委的正确领导，有勤劳智慧、勇于拼搏的全市１９３万各族人民的团结奋斗，阜新转型振兴的宏伟目标一定能够胜利实现，全市人民一定能够过上更加幸福、更加美好的生活！
